# Ecological and Geographical Analysis of the Distribution of the Mountain Tapir (*Tapirus pinchaque*) in Ecuador: Importance of Protected Areas in Future Scenarios of Global Warming

**DOI:** 10.1371/journal.pone.0121137

**Published:** 2015-03-23

**Authors:** H. Mauricio Ortega-Andrade, David A. Prieto-Torres, Ignacio Gómez-Lora, Diego J. Lizcano

**Affiliations:** 1 Laboratorio de Biogeografía, Departamento de Biología Evolutiva, Instituto de Ecología, A.C, carretera antigua a Coatepec No. 351, El Haya, 91070 Xalapa, Veracruz, México; 2 Fundación EcoCiencia, Programa para la Conservación de Especies Amenazadas de Extinción en Ecuador, Pasaje Estocolmo E2-166 and Av. Amazonas, Quito, Ecuador; 3 Museo Ecuatoriano de Ciencias Naturales, Sección de Vertebrados, División de Herpetología, calle Rumipamba 341 y Av. de los Shyris, Quito, Ecuador; 4 Centro de Modelado Científico, Eje BioCiencias, Universidad del Zulia, Maracaibo, Venezuela; 5 Master Biodiversidad en Áreas Tropicales y su Conservación, Universidad Internacional Menéndez Pelayo (UIMP)-Consejo Superior de Investigaciones Científicas de España (SCIC), Madrid, España; 6 Proyecto de conservación del Tapir Andino (Tapirus pinchaque) en la vertiente Oriental de los Andes Centrales del Ecuador; Fundación EcoCiencia, Quito, Ecuador; 7 Master en Espacios Naturales Protegidos, Fundación Fernando González Bernáldez- Universidad Autónoma de Madrid, Madrid, España; 8 Departamento Central de Investigación, Universidad Laica Eloy Alfaro de Manabí, Manta, Ecuador; Federal University of Goiás, BRAZIL

## Abstract

In Ecuador, *Tapirus pinchaque* is considered to be critically endangered. Although the species has been registered in several localities, its geographic distribution remains unclear, and the effects of climate change and current land uses on this species are largely unknown. We modeled the ecological niche of *T*. *pinchaque* using MaxEnt, in order to assess its potential adaptation to present and future climate change scenarios. We evaluated the effects of habitat loss due by current land use, the ecosystem availability and importance of Ecuadorian System of Protected Areas into the models. The model of environmental suitability estimated an extent of occurrence for species of 21,729 km^2^ in all of Ecuador, mainly occurring along the corridor of the eastern Ecuadorian Andes. A total of 10 Andean ecosystems encompassed ~98% of the area defined by the model, with herbaceous *paramo*, northeastern Andean montane evergreen forest and northeastern Andes upper montane evergreen forest being the most representative. When considering the effect of habitat loss, a significant reduction in model area (~17%) occurred, and the effect of climate change represented a net reduction up to 37.86%. However, the synergistic effect of both climate change and habitat loss, given current land use practices, could represent a greater risk in the short-term, leading to a net reduction of 19.90 to 44.65% in *T*. *pinchaque*’s potential distribution. Even under such a scenarios, several Protected Areas harbor a portion (~36 to 48%) of the potential distribution defined by the models. However, the central and southern populations are highly threatened by habitat loss and climate change. Based on these results and due to the restricted home range of *T*. *pinchaque*, its preference for upland forests and *paramos*, and its small estimated population size in the Andes, we suggest to maintaining its current status as Critically Endangered in Ecuador.

## Introduction

The mountain tapir, *Tapirus pinchaque* [1], is the smallest tapir species [2,3] and is considered to be evolutionarily distinct from its closer relatives in the Amazonian lowlands [4]. Its distribution is restricted to remnants of cloud forest and *paramo* habitats in Colombia, Ecuador and northeastern Peru, from 1,400 to 4,700 m above sea level [2,5,6]. The mountain tapir is a key species for conservation [7,8] do to its ecological role as a seed disperser and its complex history of co-evolution and adaptation to Andean environments [6,9]. Additionally, this species is currently considered as Globally Endangered by the International Union for the Conservation of Nature (IUCN). Main threats to populations in the Andes are related to habitat loss, fragmentation and hunting pressure [10]. Despite their ecological importance and conservation status, the distribution of the mountain tapir and its potential response to future climate change scenarios have not been well-evaluated, and such an understanding would have implications for its conservation at both, regional and continental scale.

In Ecuador, *T*. *pinchaque* is considered Critically Endangered [11] mainly due to fragmentation and habitat loss caused by agricultural and livestock expansion, as well as declines in populations due to wildlife trafficking (the peak period occurred from 1966 to 1971) driven by high demand from European and American zoos for new specimens, that resulted in the died of several animals [11,12]. Currently, several works have shown limited information about the mountain tapir distribution in isolated regions along the Ecuador, in areas corresponding to Cayambe-Coca National Park, Antisana Ecological Reserve, Sangay National Park, Llanganates National Park and Podocarpus Protected Areas [6,11,13,14]. However, details of its wider geographic distribution remain unclear. In this context, the National Strategy for the Conservation of Genus *Tapirus* in Ecuador highlights, as part of its action plan, shows the need to delimit the complete distribution area and ecosystem availability for *T*. *pinchaque* in the Ecuadorian Andes, for a better evaluation of its main threats and the establishment of priority conservation units [15].

An accurate delimitation of the distribution of a species holds fundamental implications for its systematics, biogeography and conservation [16]. The extent of occurrence is also a basic criterion in the establishment and allocation of a species’ conservation status at both national and international levels [8,16,17]. However, delimiting the occurrence of a species is a complex task that involves many determining factors, such as abiotic and biotic conditions, its dispersal capability and adaptability to future conditions, which are difficult to assess in the field [18].

There are marked differences in the methods available for determining the distributional limits of species [19–21], especially if we consider the growth in number of the locality records used to generate these models [22–24]. Traditionally analyses of species distribution have relied on evidence of species existence or presence records in order to delimit the ranges of species, but in most cases, records are scarce. Even when an adequate number of records exists, such records are potentially biased due to inconsistencies in accessibility of sites or differences in collection methodologies [25]. Recently, a number of sophisticated methods has been developed to estimate distributional areas by Ecological Niche Modeling (ENM), on the basis of correlating known occurrences with environmental variables. The application of technology for the modeling of ecological niches and the prediction of geographic distributions have been a useful tool in defining core areas of species diversity, studying the effect of climate change on species distributions and developing conservation strategies for species protection [26–28]. Usage of these methodologies has literally exploded in recent years, now with hundreds of papers being published every year [19,29,30], thereby improving our knowledge of species biogeography and responses to its current and future threats, especially for endangered species [31–34].

In the present study, we created a potential distribution map for *Tapirus pinchaque* in the Ecuadorian Andes, based on recently generated environmental and geospatial data to perform a Species Distribution Model (SDM). Furthermore, we evaluated the effects of several climate change scenarios, habitat loss and existing land cover, in addition to the importance of the national System of Natural Protected Areas.

## Materials and Methods

### Ethics statement

This study was carried out in strict accordance with the guidelines recommended by the National Strategy for Tapir Conservation in Ecuador, proposed by the Tapir Specialist Group/IUCN [14]. Research was conducted with geo-referenced data records from museum collections. We obtained research permits Nº 004–11 IC-FAU-DPN/MAE and MAE-DPPNO-2011–0725 from the Ecuadorian Ministry of the Environment (Ministerio del Ambiente del Ecuador), approved by Engineer S. Neptalí Rodriguez, to work with mountain tapirs in the Cayambe-Coca National Park and the Antisana Ecological Reserve. These Protected Areas (PAs), as well as surrounding areas where mountain tapirs has been previously encountered, were carefully surveyed by direct inspection looking for signs evidencing the presence of the species. No animals were captured or sacrificed over the course of this study.

### Collection of historical records

**Fig 1:**
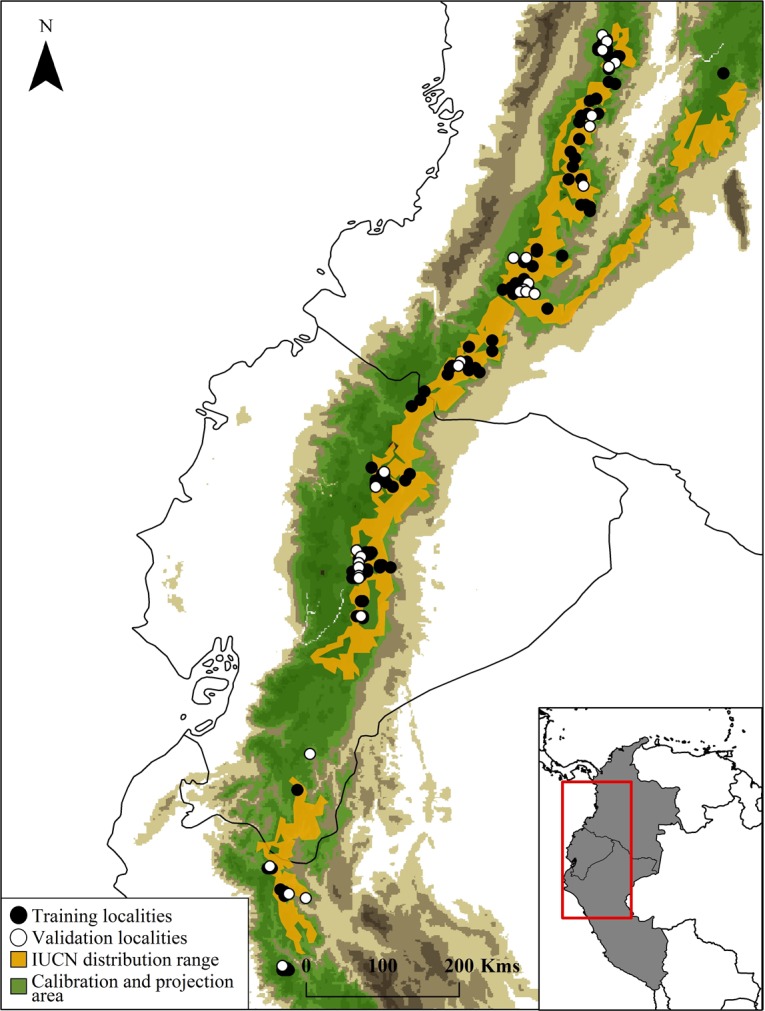
Map showing Mountain Tapir (*Tapirus pinchaque*) unique records (n = 155), overlaid with IUCN distribution and MaxEnt calibration and projection area. Training localities (black dots) and validation localities (white dots) used to generate and validate the models. Dark brown color represents area with altitudes of up 1,000 masl.

A database of available records for mountain tapir was compiled from the following three sources: 1) Collection records from the Global Biodiversity Information Facility database (GBIF; www.gbif.org) and Mammal Networked Information System (MaNIS, www.manisnet.org), 2) the database for mountain tapirs in Ecuador created by the Tapir Specialist Group (IUCN/SSC TSG-Ecuador; available under request at: www.ecociencia.org/proytapir/testphp/index.php); and 3) location records obtained from fieldwork and monitoring projects in Colombia and Peru. Each locality was geo-positioned (lat—long coordinates), using Google Earth and MapLink (http://www.maplink.com/) to correct the geographic coordinates of imprecisely recorded localities and to eliminate any inconsistencies or duplicates. Geographic coordinates were recorded in decimal degrees, based on the WGS 84 datum. We applied a buffer area between points, based on ~3,1 km^2^ home range known for the species [12], to correct the spatial bias and the high density of localities in the database. After removing duplicate information and verifying the coordinates, we had 155 historical records (Fig. 1, S1 Table), which we used to perform Ecological Niche Modeling using the Maximum Entropy algorithm [19].

### Species distribution model and validation

We modeled habitat suitability for mountain tapir using MaxEnt version 3.3.3k [19,35,36]. MaxEnt estimates the probability of distribution based on maximum entropy by applying the following principle: The expected value for each feature (*i*.*e*. climatic variables) must be equal to the empirical average value of related points with the known species presence. The algorithm performs a certain number of iterations until reaching a convergence limit, producing a map that holds values for habitat suitability ranging from 0 (unsuitable) to 1 (perfectly adequate) [36]. The program uses two data inputs: localities where the species has been recorded (presence-only data), and digital layers of the environmental conditions of a given area. The overall predictive model of distribution for the mountain tapir was generated with 80% of the locality records (training data) and the other 20% were used for evaluation (testing data). In addition, 5,000 iterations were specified to the program with no extrapolation, in order to avoid artificial projections from extreme values of the ecological variables, as such parameter is biased towards the environmental envelope of background points and occurrence data [37]. All other parameters in MaxEnt were maintained at default settings. The logistic format was used to obtain the values for habitat suitability (continuous probability from 0 to 1), which were subsequently converted to binary presence-absence values on the basis of the established threshold value, defined herein as the Fixed Omission Value 10 (FOV10; See below).

Given that ENMs do not address the historical aspects relating to species distribution (accessibility or "M" sensu BAM diagram), we used a geographical clipping based on the classification of Terrestrial Ecoregions [38], the Biogeographical Provinces of South America [39], and an altitude range limit in order to calibrate and to project the models [18,40,41]. This geographical clipping, which include the Andean region at Neotropic (from northeast Colombia to northeastern Peru) and altitude range above 1,400 m (Fig. 1), represented the species’ tolerances limits, historical barriers to dispersal, and its need for certain abiotic conditions [1,2,6,10]. For the first explorative analysis, we used the 19 climate layers from the WorldClim project [42] and assessed which variables were the most important for the model, according to the Jackniffe test calculated in MaxEnt [43]. In a second modelling exercise, we generated the species distribution using non-correlated environmental variables (*r* < 0.8) in combination with the most relevant environmental variables identified in the first approach. These additional steps allowed us to reduce over-fitting of the generated distribution models [44,45]. We used the same bioclimatic datasets to generate models for present and future scenarios (See below).

We evaluated the performance of the MaxEnt model by calculating the commission and omission errors [46], the Area Under the Curve (AUC) of the Receiver Operating Characteristic (ROC) curve [19,37], as well as by the partial ROC curves test [47]. The aforementioned criterion is used to solve problems associated with the AUC, avoiding an inappropriate weighting of the omission and commission components in the analysis [47,48]. We calculated partial AUCs using the Tool for Partial-ROC V. 1.0. [49], using 20% of the original data for the independent model evaluation. We presented the partial ROC results as the ratio of the AUC model to the null expectation ["AUC ratio"; 48]. The statistical significance of AUCs was assessed by bootstrapping manipulations and by comparison with the null expectations. Resampling represented the assignment of 50% of the points from the overall pool of data with replacement values 1,000 times. Significance (*e*.*g*. elevation above the line of null expectation) was assessed by ranking the observed values (calculated AUC) with the values of the pseudoreplicates, following the proposal of Peterson *et al*. [48].

Finally, to aid model validation and interpretation, suitable areas were distinguished from unsuitable areas by setting a decision threshold value that represented a positive prediction for species presence. There is not a set rule to determine such thresholds, as its selection commonly depends on the dataset used or the objectives of the model, and varying from species to species [28]. For this study, we decided to use values equal to the Fixed Omission Value 10 (FOV). This threshold can be interpreted ecologically by identifying those pixels predicted with this value to be at least as suitable as those pixels where the species has been previously recorded, allowing the omission of ~10% of presences points [28,45]. For our purposes, this threshold allows us to evaluate the species' distribution by minimizing commission errors in our final binary maps.

### Ecological niche modeling in scenario of future global climate change

Despite the potential problems associated with the use of global climate change scenarios at local scales [50], such approaches are useful in demonstrating potential tendencies and future threats to species. Global Climate Models (GCMs) describes future scenarios by considering several components of radioactive forcing used for modeling climate and atmospheric chemistry, such as emissions of greenhouse gases, air pollutants and land use. The potential distribution of *T*. *pinchaque* was assessed for two Representative Concentration Pathway scenarios (RCP 4.5 / RCP 8.5) developed by three different sources: (a) Commonwealth Scientific and Industrial Research Organization and Bureau of Meteorology (ACCESS 1.0); (b) Model for Interdisciplinary Research On Climate (MIROC5); and (c) the Met Office Hadley Centre and Instituto Nacional de Pesquisas Espaciais (HadGEM2-ES). The GCMs were downloaded from the WorldClim website (http://www.worldclim.org/cmip5_30s) as digital layers based on the same bioclimatic variables used to generate the species distribution model.

The climatic models used here represent two moderate scenarios of emission concentrations for each greenhouse gas (*e*.*g*. CO_2_, methane, nitrous oxide, etc.), which serve as proxies for a wide range of scientific and socioeconomic data, such as population growth, air pollution, land use and energy sources [51,52]. In a general context, the RCP 8.5 scenario represents a higher predicted greenhouse gas emissions compared with RCP 4.5, although both assume increasing human population, relatively slow income growth and modest improvements in technology and energy intensity, leading to a higher demand for energy and increasing greenhouse gas emissions in the long-term considering an absence of climate change mitigation policies [53]. We predicted the persistence of mountain tapir’s ecological niche in 2050, since the species faces multiple conservation problems that may threaten its survival in the short term. More distant scenarios would be subject to higher levels of uncertainty [50]. Thus, we generated maps representing the potential distribution of mountain tapir in 2050, under the RCP 4.5 (optimistic) and RCP 8.5 (pessimistic) climate change scenarios. Six maps were obtained for the modeled future forecasts for *T*. *pinchaque* (from two scenarios and three sources), however, only ACCESS 1.0 RCP4.5 and RCP8.5 maps were shown (See “Results”).

To assess the shifts in the relationships between probability of suitability and altitude we explored and compared differences in the centroids for the scattered plots of the models. Because the data from suitability probabilities are strictly bounded, the variance is non-constant and errors are non-normal, we fitted generalized linear models (GLMs) for proportion data to assess the variable of altitude between scenarios, along with a two-parameter logistic function that is the equivalent of an analysis of covariance with binomial errors [54]. To assess the trends in the data, afterwards we fitted a Generalized Additive Model (GAM) to add a non-parametric smoothed line to the plots. GLMs and GAM analyses were realized using the R software [55] and the *mgcv* library.

### Spatial analysis of the mountain tapir distribution in Ecuador

The models obtained were clipped to the national scale of Ecuador for the subsequent spatial analyses, which included an assessment of the impacts of deforestation, ecosystem availability, and PAs. To assess the effect of deforestation, we used a vegetation land cover map classified by ecosystems for continental Ecuador [56]. In order to assess the effect of habitat loss, we only considered two categories of natural forest and perturbed areas, with the latter category containing urban areas, deforested areas, farming areas and pastures for cattle ranching [45].

In the first approach, the importance of each ecosystem was assessed simply by extracting the suitable areas (km^2^) generated by the model and sorting them by area size in descending order, from the largest ecosystem area to the smallest. In a second approach, we divided the total suitable area of the model by the total available area per ecosystem in Ecuador, resulting in the relative ecosystem importance (REI = model suitability area / ecosystem availability area). These values ranged from 0–1 and tends towards 1 when the total available area is equal to the suitable area, which is calculated by the model for each ecosystem. The total area per ecosystems was obtained from maps produced by the Ministry of Environment of Ecuador [56].

We also evaluated the importance of Ecuadorian PAs for the obtained models, using layers downloaded from ProtectedPlanet.net [57]. Additionally, we compared our results with the extent of occurrence calculated from a geometric convex hull polygon that resulted from the union of all verified localities. The use of a polygon could lead to the underestimation and a restriction in the range of occurrence of the species, especially when occurrence is expected to be found for additional unverified localities. However, this alternative method was applied as it is commonly used to evaluate and compare the extension of predicted presence for threatened species [8,58]. All spatial analyses and map algebra were carried out with ArcMap 10.2.2 software, and the convex hull polygon was calculated using the “minimum bounding geometry” tool in ArcTool Box [59]. The grid cell resolution, or pixel size, was 0.0083 degrees, corresponding to ~1 km^2^ in each raster.

## Results

### Ecological niche and species distribution model for *T. pinchaque*

**Fig 2:**
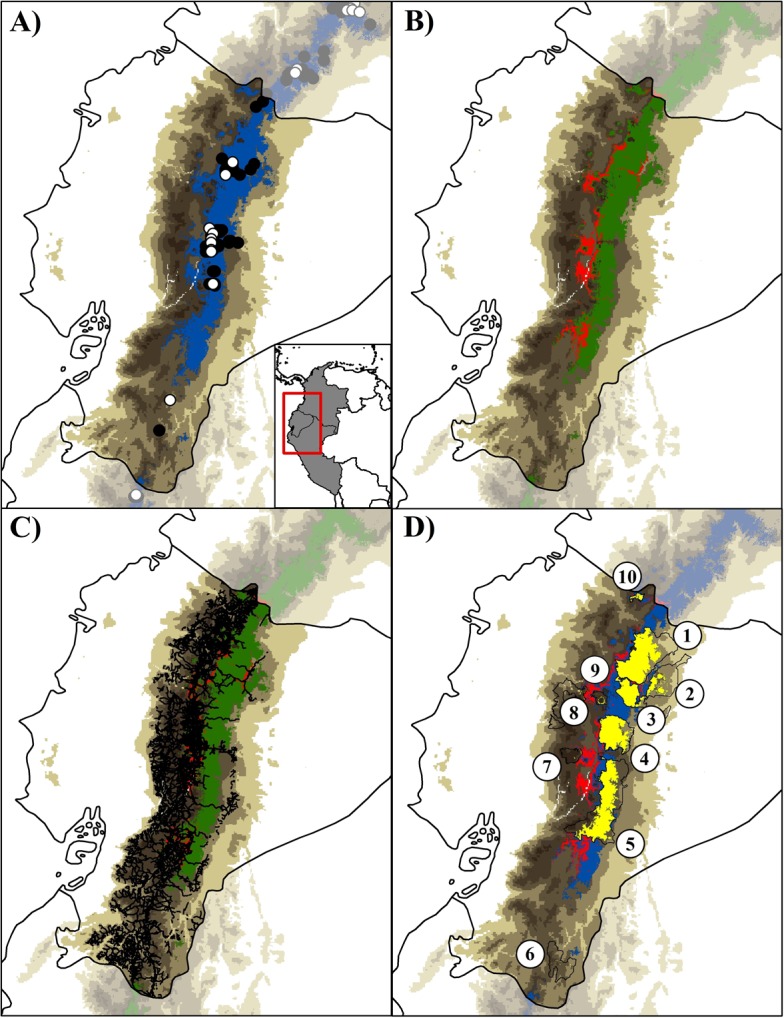
Potential distribution model of *Tapirus pinchaque* in Ecuador. (A) Potential distribution model is shown with the threshold value of Fixed Omission Value 10 (FOV10, dark blue); (B and C) Remnant potential distribution model with natural forests (green areas), perturbed areas (red) and principal roads in Andes of Ecuador (black lines); (D) Remnant potential distribution model predicted for Protected Areas of Ecuador (yellow areas bordered by black) and perturbed areas (red). Training localities (black dots) and validation localities (white dots) used to generate models are shown in (A). Numbers in (D) correspond to: Cayambe—Coca National Park (1); Sumaco Napo-Galeras National Park (2); Antisana Ecological Reserve (3); Llanganates National Park (4); Sangay National Park (5); Podocarpus National Park (6); Chimborazo Faunistic Reserve (7); Los Illinizas Ecological Reserve (8); Cotopaxi National Park (9); and El Angel Ecological Reserve (10). Note an important reduction (~17%, in green) in the western Andes of the best-predicted potential distribution model (FOV10 threshold) when filtered to only include areas of natural forests (B), and a reduction of 52% when it was filtered to only include Protected Areas in Ecuador (D). Dark brown color represents area with altitudes of up 1,000 masl. The model was generated with the no-correlated environmental variables (Table 1).

The model showed a high success-rate for the AUC-test (0.910) and AUC ratio (1.29 ± 0.16; *p* < 0.05) values, with a 9.7% rate of omission and a FOV10 (logistic threshold value) of 0.250. The environmental variables used and their percentage contribution to the model are shown in the Table 1. The predicted suitability area for mountain tapir was ~52,000 km^2^ along the Andean region from Colombia to northeastern Peru, with an estimated area of ~21,700 km^2^ (~42% of the model) restricted to the Ecuadorian Andes (Fig. 2A). In contrast, the extent of occurrence for mountain tapir in Ecuador is represented by a convex hull polygon with ~20,500 km^2^ (Table 2).

**Table 1:** Summary of the selected, no-correlated, environmental variables with relative contributions (%) to the model of *Tapirus pinchaque*.

Abbreviation	Environmental Variable	Percentage of contribution
Bio 08	Mean Temperature of Wettest Quarter	37.70
Bio 14	Precipitation of Driest Month	17.60
Bio 19	Precipitation of Coldest Quarter	15.00
Bio 07	Temperature Annual Range (BIO5-BIO6)	6.50
Bio 03	Isothermality (BIO2/BIO7) (* 100)	5.60
Bio 02	Mean Diurnal Range	5.00
Bio 12	Annual Precipitation	4.50
Bio 04	Temperature Seasonality (standard deviation *100)	3.90
Bio 17	Precipitation of Driest Quarter	2.20
Bio 01	Annual Mean Temperature	2.10

**Table 2:** Potential distribution and ecological niche models for *Tapirus pinchaque*, with percentage loss of extent of occurrence under differing global climate models (RCP 4.5/ RCP 8.5 scenarios), natural forest and Protected Areas (PAs) in the tropical Andes of Ecuador.

Model	Area (~km^2^)	%
**Present potential distribution area**	21,729	100.00
Area of the model within natural forests	17,990	82.79
Area of the model within PAs	10,738	49.42
Remnant model within PAs and natural forests	10,473	48.20
**IUCN distribution map**	20,610	94.85
**Extent of occurrence (convex hull polygon)**	20,421	93.98
**Models under RCP 4.5**		
**ACCESS 1.0**		
Potential distribution area	16,990	78.19
Area of the model within natural forests	14,671	67.52
Area of the model within in PAs	9,184	42.27
**MIROC 5**		
Potential distribution area	21,071	96.97
Area of the model within natural forests	17,404	80.10
Area of the model within in PAs	10,305	47.43
**HadGEM2-ES**		
Potential distribution area	16,271	74.88
Area of the model within natural forests	14,019	64.52
Area of the model within in PAs	9,189	42.29
**Models under RCP 8.5**		
**ACCESS 1.0**		
Potential distribution area	13,502	62.14
Area of the model within natural forests	12,028	55.35
Area of the model within in PAs	7,888	36.30
**MIROC 5**		
Potential distribution area	20,889	96.13
Area of the model within natural forests	17,170	79.02
Area of the model within in PAs	10,519	48.41
**HadGEM2-ES**		
Potential distribution area	14,199	65.35
Area of the model within natural forests	12,540	57.71
Area of the model within in PAs	8,337	38.37

### Impacts of deforestation and ecosystem availability on the distribution model in Ecuador

The predicted and remnant areas of the potential distribution model for the mountain tapir in the Ecuadorian Andes are detailed in Tables 2 and 3. Intensive deforestation reduced by ~17% the predicted extent of occurrence for *T*. *pinchaque* (Table 2). Habitat loss was the most pronounced along the highlands of the Central Andes and near to the roads that connect the large cities of the Azuay, Chimborazo, Tungurahua, Cotopaxi, Pichincha and Carchi provinces (Fig. 2B-C). Additionally, a significant reduction of ~52% (11,256 km^2^) of the potential distribution area for *T*. *pinchaque* was identified when we considered only remnants of natural forest within the limits of PAs (Fig. 2D; Tables 2–3). A total of 30 records (52,6% of Ecuadorian data) were reported within the limits of PAs.

**Table 3:** Potential distribution model of the Mountain Tapir, *Tapirus pinchaque*, in km^2^ (percentages), predicted for Protected Areas in the Ecuadorian Andes.

Protected area	Model in PA	Remnant forest in PA
1. Cayambe—Coca National Park	3,330 (15.33%)	3,206 (14.75%)
2. Sumaco Napo-Galeras National Park	476 (2.19%)	476 (2.19%)
3. Antisana Ecological Reserve	1,234 (5.68%)	1,222 (5.62%)
4. Llanganates National Park	2,084 (9.59%)	2,045 (9.41%)
5. Sangay National Park	3,386 (15.58%)	3,323 (15.29%)
6. Podocarpus National Park	7 (0.03%)	7 (0.03%)
7. Chimborazo Faunistic Reserve	16 (0.07%)	13 (0.06%)
8. Cotopaxi National Park	43 (0.2%)	40 (0.18%)
9. Los Illinizas Ecological Reserve	29 (0.13%)	8 (0.04%)
10. El Angel Ecological Reserve	133 (0.61%)	133 (0.61%)
**Total predicted model in Protected Areas**	**10,738 (49.42%)**	**10,473 (48.2%)**

Ordered numbers correspond to the labeled identities in Fig. 2D.

A total of 26 ecosystems in the Ecuadorian Andes were found within the area generated by potential distribution model for mountain tapir; ten of them encompass ~98% of its total area (Table 4). In the first approach, the most extensive ecosystems were the herbaceous *paramo* (5,601 km^2^; ~32% of the model), the northeastern Andes montane evergreen forest (4,208 km^2^; ~24%), the northeastern Andes lower montane evergreen forest (2,724 km^2^; ~15%) and the northeastern Andes upper montane evergreen forest (1,674 km^2^; ~9%), which cover more than 80% of the distribution area predicted by the model. In the second approach, we analyzed the relative ecosystem availability (REI), where only two ecosystems had values greater than 0.90 (the northeastern Andes upper montane evergreen forest and the Sumaco vulcano evergreen herbaceous and shrubland *paramo*), three ecosystems ranged from 0.60 to 0.89, ten ecosystems ranged from 0.10 to 0.59, and 11 ecosystems ranged from 0.001 to 0.09 (Table 4). However, only the northeastern Andes upper montane evergreen forest (REI = 0.91) and the northeastern Andes montane evergreen forest (REI = 0.80) had values that agreed with the importance rank of ecosystem availability assessed by the first approach. On the other hand the Sumaco vulcano evergreen herbaceous ecosystem had available areas that fully matched with the suitability areas predicted by the model (REI = 1), although their overall area available in Ecuador is small (~4 km^2^).

**Table 4:** Ecosystem availability (km^2^) and suitability areas according to the potential distribution model (km^2^) and relative importance of ecosystem availability (REI = suitability/availability) calculated for *Tapirus pinchaque* in Ecuador.

No	Ecosystem [56]	Ecosystem availability in Ecuador (Km^2^)	Suitability area (Km^2^)	Model importance (%)	Cumulative importance (%)	REI*
1	Herbaceous *paramo*	12,305	5,601	31.50	31.50	0.46
2	Northeastern Andes montane evergreen forest	5,276	4,208	23.66	55.16	**0.80**
3	Northeastern Andes upper montane evergreen forest	2,989	2,724	15.32	70.48	**0.91**
4	Southeastern Andes montane evergreen forest	4,741	1,674	9.41	79.89	0.35
5	Shrubland evergreen and montane herbaceous *paramo*	2,679	1,230	6.92	86.81	0.46
6	Southeastern Andes upper montane evergreen forest	1,475	941	5.29	92.10	**0.64**
7	Cushion and *Espeletia paramo*	543	363	2.04	94.14	**0.67**
8	Northeastern Andes lower montane evergreen forest	4,941	360	2.02	96.16	0.07
9	Southeastern Andes lower montane evergreen forest	2,462	162	0.91	97.08	0.07
10	Subnival herbaceous and shrubland *paramo*	789	98	0.55	97.63	0.12
11	Northern Andes montane evergreen shrubland	641	73	0.41	98.04	0.11
12	Western Andes upper montane evergreen forest	1,617	69	0.39	98.43	0.04
13	Montane-lake herbaceous *paramo*	140	57	0.32	98.75	0.41
14	Condor-Kutukú cordillera montane evergreen forest	1,181	52	0.29	99.04	0.04
15	Evergreen forest *paramo*	96	47	0.26	99.30	0.49
16	Subnival ultra-wet herbaceous *paramo*	201	41	0.23	99.53	0.20
17	Subnival humid herbaceous *paramo*	100	20	0.11	99.65	0.20
18	Condor-Kutukú cordillera lower montane evergreen forest	2,985	13	0.07	99.72	0.00
19	Upper montane humid herbaceous *paramo*	425	11	0.06	99.78	0.03
20	Condor cordillera shrubland evergreen and montane herbaceous *paramo*	284	9	0.05	99.83	0.03
21	Southern Andes lower montane-lake herbaceous	23	8	0.04	99.88	0.35
22	Northern Andes valleys of Semi-deciduous forest and shrubland	686	7	0.04	99.92	0.01
23	Southeastern Andes foothill evergreen forest	1,289	6	0.03	99.95	0.00
24	Western Andes montane evergreen forest	3,806	4	0.02	99.97	0.00
25	Sumaco vulcano evergreen herbaceous and shrubland *paramo*	4	4	0.02	99.99	**1.00**
26	Catamayo-Alamor evergreen upper montane forest	197	1	0.01	100.00	0.01
	**Total**	**51,875**	**17,783**	**100**	**100**	**0.34**

Ecosystems are sorted in descending order from the largest to smallest representative area as calculated by the model. The first 10 ecosystems represent ~98% of the distribution model.

*Values in REI ranged from 0–1, tending towards 1 when the total available ecosystem area was equal to the model suitable area.

Bold numbers represent ecosystems with availability of over 60% in Ecuador. Perturbed and without data areas were removed from the analysis.

### Future scenarios of climate change

**Fig 3:**
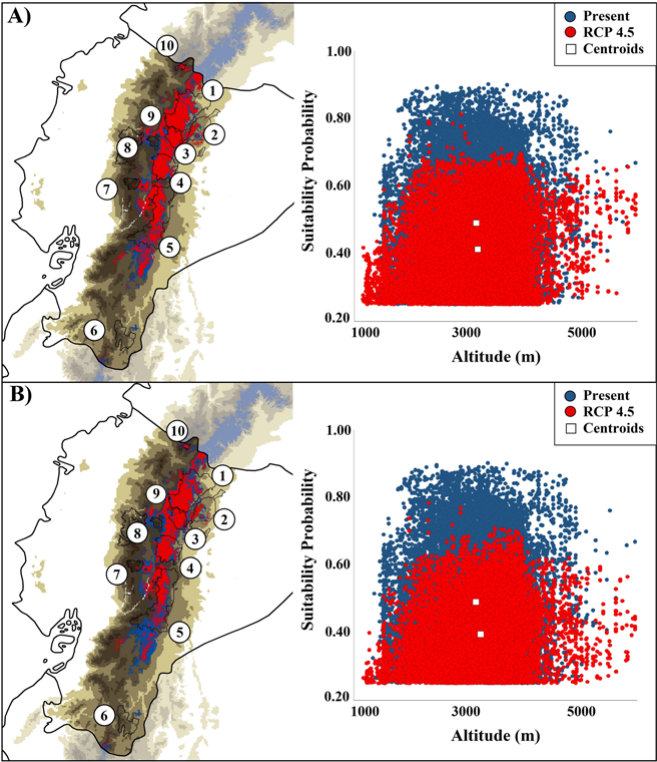
Potential ecological niche model of *Tapirus pinchaque* in two future scenarios of climate change based on the Global Climate Models of ACCESS 1.0. Potential ecological niche model for the year 2050, under (A) RCP 4.5 (optimistic) and (B) RCP 8.5 (pessimistic) climatic change scenarios. Numbers correspond to those referred to for Protected Areas estimation in Fig. 1. Note a reduction (~22–38%, in red) of the best-predicted potential distribution model (FOV10 threshold). In both scenarios, suitability areas for *Tapirus pinchaque* tend to critically reduce, especially in the southern regions. Shifts are depicted from their centroids in the ecological niche-space.

**Fig 4:**
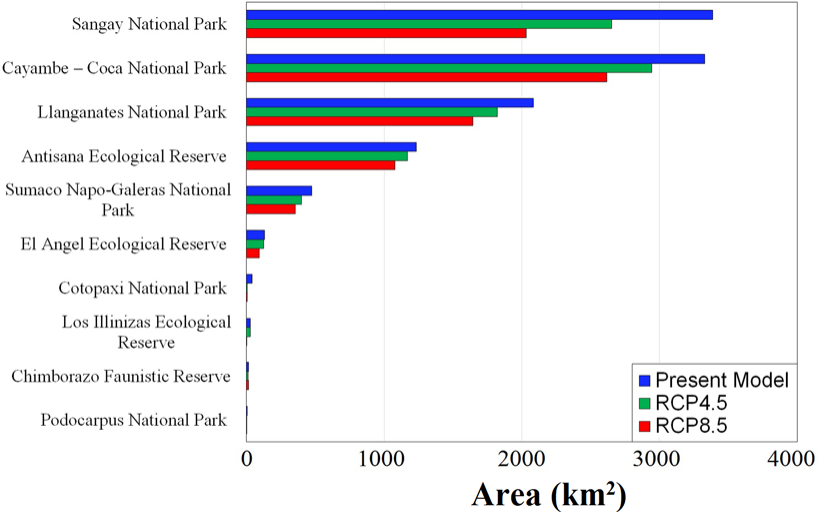
The role of Protected Areas in two future scenarios of climate change (based in ACCESS 1.0). Potential distribution model in the year 2050, under RCP 4.5 (optimistic) and RCP 8.5 (pessimistic) climatic change scenarios. In both scenarios, we observed a reduction in the predicted geographic range for *Tapirus pinchaque*.

Potential geographic ranges projected for mountain tapir in Ecuador under different climate change scenarios are presented in Fig. 3. Reductions in the predicted extent of occurrence caused by habitat loss and the representation of the range encompassed by PAs in different climate change scenarios are detailed in Table 2. The suitability areas for mountain tapirs were predicted to reduce in extension, mainly in the lowlands of central and southeastern Ecuadorian (Fig. 3). Climate change will likely be an influencing factor in the distribution of the mountain tapir, since changes associated with climate represented a net reduction in extent of occurrence ranging from 3.03 to 25.12% in the RCP 4.5 scenario and from 3.87 to 37.86% in the RCP 8.5 scenario (Table 2). The PAs harbor between 42.27 to 47.43% of the distribution area in RCP 4.5 scenario and between 36.30 to 48.41% in RCP 8.5 (Table 2). These patterns were found to be consistent even upon comparing the three different global climate models (GCMs). Only the models from MIROC5 laboratory seemed to be less sensitive, representing a less restricted suitability area given scenarios of climate change (Table 2). Additionally, the projection models for the year 2050, under both optimistic and pessimistic climatic change scenarios, tended to result in a critical reduction in suitable area for *T*. *pinchaque* within the limits of several PAs (Fig. 4), including the Cotopaxi National Park (~87%), Podocarpus National Park (~64%), Los Illinizas Ecological Reserve (~50%), and Sangay National Park (~31%). Meanwhile in other PAs, the reduction of the predicted geographic range was less pronounced.

**Fig 5:**
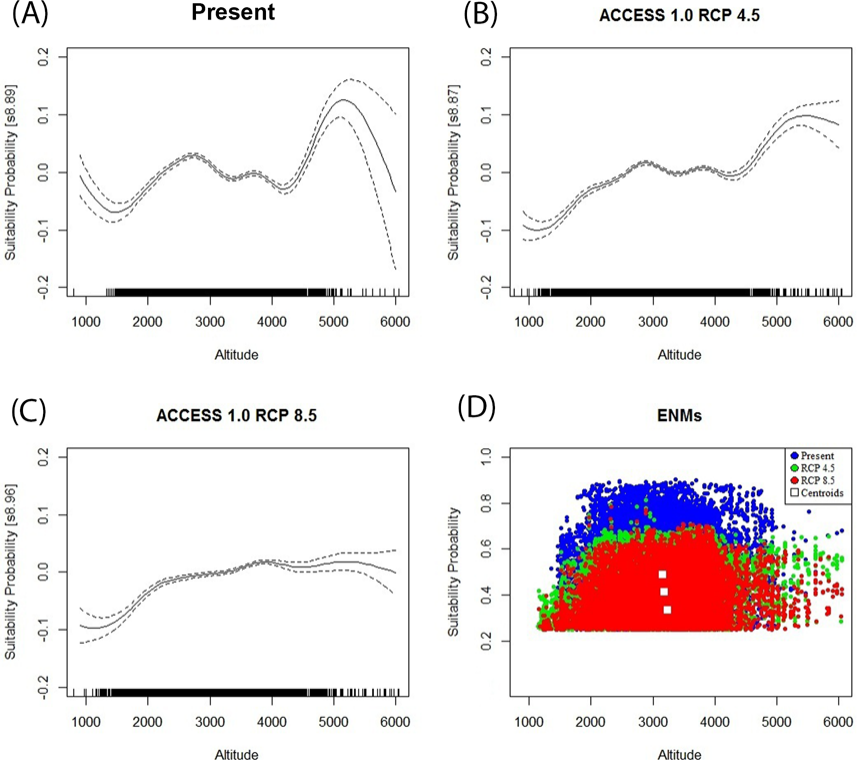
Generalized Additive Models (A-C) and scatter plot for the relation between suitability probability and altitude, in climate change scenarios (D). Non-parametric smoothers in GAM showing the multiple humped relationship between suitability probability and altitude, highlighting the possibility of minimum thresholds of altitude over ≈ 3,000 m (A-C). The etchings (small black lines) on the *x* axis in GAMs (A-C) indicate the density of probabilistic values located along the altitude, whereas centroids for the scattered data are represented as squares in (D).

Finally, we found that for future scenarios, the suitable areas predicted per model tended to significantly increase with the altitude (Figs. 3 and 5). On average, there is a shift of ~56 m in altitude between the range of the present scenario (3,156 ± 664 m) and the two climate change scenarios (ACCESS 1.0 RCP 4.5 = 3,183 ± 705 m; RCP 8. 5 = 3,240 ± 692 m). Furthermore, a significant reduction in mean habitat suitability was predicted for the climate change scenarios, RCP 4.5 (ACCESS 1.0: suitability probability = 0.412 ± 0.108; 0.250–0.812) and RCP 8.5 (ACCESS 1.0: suitability probability = 0.335 ± 0.103; 0.250–0.784), when compared with the present scenario (suitability probability = 0.490 ± 0.154; 0.250–0.902). Shifts in suitability probability and altitude are represented by the centroids of a scattered data plot in Figs. 3 and 5. Regression models for suitability probabilities (SP) follow a two-parameter logistic function: *y* = $\frac{e^{a+bx}}{{1+e}^{a+bx}}$; whereas linearized coefficients (*a+bx*) for the present scenario were (PS) = –0.01618 + (-0.00000727 [altitude]), RCP 4.5 (PS) = –0.5979 + (-0.0000764 [altitude]) and RCP 8.5 (PS) = –0.7283 + (-0.0000925 [altitude]). Curvature of non-parametric smoothers in GAM showed a multiple humped relationship between suitability probability and altitude, highlighting the possibility of minimum thresholds of altitude of ~3,000 masl (Fig. 5A-C). Overall, deviance explained by GAMs ranged from 2 to 4%, and adjusted *r* ^2^ ranges varied from 0.019 to 0.039 in the models.

## Discussion

In this study we presented potential distribution models for *Tapirus pinchaque* in the Ecuadorian Andes and assessed the effects of climate change under two future scenarios in 2050. The geographic implications of these models are discussed in the context of Ecuadorian System of Natural Protected Areas where we propose the ENM approach as an important component of conservation research and planning, especially relevant for mountain species facing habitat loss and scenarios of climate change.

### Species distribution model, impacts of deforestation and ecosystem availability

Deforestation and the expansion of the agricultural frontier are both the most important factors affecting habitat and biodiversity loss in the Neotropics [60–64]. In Ecuador, in addition to a long history of habitat modification that dates back to pre-Columbian times [65–67], a more recent accelerated fragmentation and degradation of the landscape extension have occurred, leading to a dramatic reduction in over 55% of the natural vegetation cover [27,56]. Such regional and large-scaled habitat modifications may change local climate patterns, even those of well-preserved areas, and thus affect species, especially those with restricted geographic ranges, such as the mountain tapir. The suitability model showed an important reduction (~17%) in area when we considered the effect of habitat loss at a national scale, although only a 1.22% reduction occurred inside PAs (Tables 2–3). Human land uses may be particularly impactful in the Andes, where anthropogenic activities and the presence of roads may create solid barriers that prevents northward species migrations [68], possibly pushing the mountain tapir to the edge of its distributional area and fragmenting the connectivity of the predicted suitability areas.

From the list of 26 ecosystems in the Ecuadorian Andes related to the presence of *T*. *pinchaque*, ten ecosystems harbor ~98% of the model area. Of these ecosystems, we identified several as priorities for conservation planning, including the herbaceous *paramo*, the northeastern Andes montane evergreen forest, the northeastern Andes upper montane evergreen forest, and the northeastern Andes montane evergreen forest, as they represented over the 75% of area predicted by the model. On the other hand, considering the relative ecosystem availability, these areas also included the southeastern Andes upper montane evergreen forest, the Cushion and *Espeletia paramo*, and the Sumaco vulcano evergreen herbaceous and shrubland *paramo*. All of these ecosystems are associated with Andean highlands and are highly threatened by human activities, including the expansion of cattle ranching and urban settlements that remove vegetation cover and thereby threaten or restrict the mountain tapir’s habitat [12]. Future conservation efforts should concentrate on reducing habitat destruction and restoring the identified natural habitats, especially considering the restricted home range and reduced population size estimate for this species [12].

### Impacts of climate change

After modeling the potential extent of occurrence under two climate change scenarios by 2050, our results revealed that bio-climatically suitable areas for this species will decline in Ecuador (Table 2, Figs. 3–5). The main conclusions related to this are: a) an upward shift of ideal climatic conditions towards higher elevations, b) an overall reduction in the area occupied by the species, and c) the potential disappearance of suitable areas from the mountain tapir’s current distribution range (Table 2). These patterns were found consistently across the three different global climate models (GCMs).

Mountain species are especially sensitive to climate change, since their ecological niche potentially increases or decreases in altitude, thereby restricting the available surface area that they may inhabit [69–71]. Our results reinforce the widely accepted point of view that significant changes in the ranges of mountain biota will occur during the twenty-first century as a result of climate change [72–74], which could threaten or lead to the extinction of many species in the Andean mountain range. For *T*. *pinchaque*, for example, we observed that the predicted probability of occurrence decreased steadily along an altitudinal gradient, with a clear restriction in elevation from 2,500 to 4,500 masl in future climate change scenarios (Figs. 3 and 5). These patterns are consistent with other studies focusing on Andean species that have predicted an migration upslope in response to global warming [68,73]. Species may respond to climate change by shifting bioclimatic niches to different elevations or latitudes or by moving to areas where temperatures are optimal for their survival. As regional temperatures increase, certain species may not persist unless they are able to move to higher elevations [75–77]. The mountain tapir is considered a key species due to its complex history of co-evolution and adaptation in the Andes and its important ecological role as seed disperser [6,9]. In this context, if rapid modifications in the structure and composition of plants related to the mountain tapir are provoked by climate change in the Andes, these threats may also lead to significant impacts on the ecological niche of the species, and its abundance and longevity.

The adaptive potential of the species is determined by its own evolutionary rate and ability to respond to rapid environmental change in a relatively short period of time. Unfortunately, long-lived organisms, such as the mountain tapir, may not evolve rapidly due to demographics restrictions and the delay in onset of sexual maturity [78,79]. If the pace of climate change under scenarios of global warming exceeds the species’ ability to migrate, the mountain tapir should be considered extremely vulnerable throughout its entire range [68,73]. Climate change may represent a high extinction risk for the mountain tapir in Ecuador, especially if we consider the synergistic effects of habitat loss and habitat exclusion stemming from cattle ranching and potential diseases [12,80].

### System of natural PAs

The PAs are form part the most common strategies in reducing global biodiversity loss and are central to the aims of almost all conservation policies [27,81]. However, Ecuadorian Protected Areas represent the ~49% of the area of potential distribution for mountain tapir, encompassing the Sangay, Cayambe-Coca and Llanganates National Parks, which represent the most suitable areas (Table 3; Figs. 2 and 4). All the PAs tends to reduce the total suitable range area for this species, considering future scenarios of climate change, especially towards the Sangay National Park (Fig. 4). Herein we demonstrated that most of perturbed areas are located along northern-central Andes and the foothills of the southeastern Andes of Ecuador, where the expansion of the agricultural frontier, cattle pastures, wood extraction, illegal mining and infrastructure development are advancing fast, especially in zones nearby to Protected Areas (Fig. 2D). This evidence agrees to the UICN evaluation criteria to categorize this species as Endangered. On the other hand, in the northern and central Andes a less pronounced conservation gap exists when compared with southern Ecuador. Five National Parks (Cayambe-Coca, Sumaco Napo-Galeras, Llanganates, Sangay, and Cotopaxi) and one Ecological Reserve (Antisana) are PAs located in the central and northern regions, which could provide a better opportunity to conserve viable mountain tapir populations. The persistence of PAs in Ecuador will play an important role in preventing environmental degradation in the central and northern portion of the mountain tapir distribution, where populations along the southern Ecuador are more vulnerable due to less PAs.

In scenarios of global warming, the current PAs will preserve ~36 to 48% of the extent of occurrence predicted by the models for mountain tapirs. Given the potential reduction in areas and the vulnerability of *T*. *pinchaque* in scenarios of climate change, it is important to propose an assessment of mountain tapir populations within the National PAs. Potential ecological corridors connecting El Angel—Cotopaxi Ecological Reserves, and the Cayambe Coca—Sumaco Napo Galeras—Antisana National Parks, will be useful providing local and regional connections in the northern portion of the mountain tapir’s distributional range, and throughout the central-southern area between the Sangay and Llanganates National Parks [55]. Few studies have focused on the identification of priority areas for the conservation of large mammals and ecosystems in continental Ecuador [27,82,83]. Such studies may provide key tools in assessing potential conservation areas for a variety of species in order to define conservation goals and create corridors in a more realistic framework, therefore increasing the efficiency of conservation strategies in the ecologically important Andean region of Ecuador.

### Implications for conservation and future perspectives

Highland Andean ecosystems are home to a diverse array of unique wildlife and are important providers of ecosystem services to the inhabitants of this region [84]. Considering the *T. pinchaque’*s restricted home range, high fidelity to upland forests and *paramos*, and the small population size assessed for Andes of Ecuador [12], the conservation of the natural habitats is crucial for its survival. Even though we only examined the distribution of the mountain tapir in Ecuador, our results begin to elucidate how valuable such information is for planning or tapir conservation. The models forecast a rapid decline in population of the mountain tapir in the Ecuadorian Andes, principally attributed to a decrease in occupancy area, range of occurrence and loss of habitat area or quality [11,12,15]. On the other hand, the synergistic effect of these factors combined with climate change may represent a prominent risk in the short-term, especially along the southern portion of the mountain tapir’s distribution, where few PAs are located (Figs. 3 and 4). In addition, it is important for future research to examine the effects of increasing human pressures and cattle ranching, as well as its impact on upland forests and *paramos*. The proposal of new PAs should consider the protection of key species habitat using science based methods in order to increase regional connectivity and complement the current network of PAs [27]. Our results, are according to the national categorization of Critically Endangered proposed for the mountain tapir in Ecuador [11].

Very little is known about the synergistic effects of diseases transmission and resource competition (between tapirs and cattle) on population declines of the mountain tapir, in addition to the effects of habitat fragmentation and the expansion of agricultural frontier. Therefore, future conservation efforts should concentrate on these topics, as well as on genetic studies of populations in the Andean region to evaluate their genetic structure, phylogenetic relationships and population dynamics of mountain tapirs. An integrative approach should be used to define conservation priorities units and to design ecological corridors along the mountain tapir’s distributional range.

Like other large charismatic species in South America such as the jaguar, condor, Andean bear, etc., the conservation of mountain tapirs may ensure that many other sympatric species also be benefitted. The mountain tapir has a unique history of co-evolution with the Andean landscape that should be promoted by local governments and non-governmental organizations as a key regional symbol in conservation campaigns, educational initiatives and scientific research.

## Supporting Information

S1 Table Historical records of *Tapirus pinchaque* used to generate the species distribution model. Geographic coordinates are provided in decimal degrees, based on the WGS 84 datum.
(DOCX)
