# Supplementary material for: Ecological and Geographical Analysis of the Distribution of the Mountain Tapir (Tapirus pinchaque) in Ecuador: Importance of Protected Areas in Future Scenarios of Global Warming
Source: PLoS One. 2015 Mar 23;10(3):e0121137. doi: 10.1371/journal.pone.0121137 (PMC4370470; doi:10.1371/journal.pone.0121137)
Supplement: S1 Table — Geographic coordinates are provided in decimal degrees, based on the WGS 84 datum. (DOCX) [file pone.0121137.s001.docx]

**Supporting Information**

| **S1 Table. Historical records of *Tapirus pinchaque* used to generate the Species Distribution Model.** Geographic coordinates are provided in decimal degrees, based on the WGS 84 datum. Source: **GBIF** = Global Biodiversity Information Facility database; **MaNIS** = Mammal Networked Information System; **IUCN/SSC TSG-Ecuador =** Database for mountain tapir in Ecuador created by the Tapir Specialist Group; **Field Work** = Localities records obtained from fieldwork monitoring; **UTPL** = Universidad Técnica Particular de Loja (Rodrigo Cisneros, comm. pers.). | | | | |
| --- | --- | --- | --- | --- |
| **COUNTRY** | **STATE /PROVINCE** | **LONGITUDE** | **LATITUDE** | **SOURCE** |
| Colombia | Carchi, Tulcán | -77.643299 | 0.677293 | Field Work |
| Colombia | Cauca, Purace | -76.426633 | 2.252293 | Field Work |
| Colombia | Cauca, Purace | -76.451633 | 2.193960 | Field Work |
| Colombia | Cauca, San Sebastian | -76.701633 | 1.877293 | Field Work |
| Colombia | Cauca, Santa Rosa | -76.584966 | 1.835627 | Field Work |
| Colombia | Cauca, Sotara | -76.584966 | 2.252293 | Field Work |
| Colombia | Cundinamarca, Chipaque | -74.100000 | 4.416667 | Field Work |
| Colombia | Huila, La Plata | -76.309966 | 2.343960 | Field Work |
| Colombia | Huila, La Plata | -76.318299 | 2.335627 | Field Work |
| Colombia | Huila, La Plata | -76.018299 | 2.268960 | Field Work |
| Colombia | Huila, Palestina | -76.184966 | 1.660627 | Field Work |
| Colombia | Huila, San Agustin | -76.451633 | 1.993960 | Field Work |
| Colombia | Huila, San Agustin | -76.518299 | 1.960627 | Field Work |
| Colombia | Huila, San Agustin | -76.401633 | 1.952293 | Field Work |
| Colombia | Huila, San Agustin | -76.568299 | 1.943960 | Field Work |
| Colombia | Huila, San Agustin | -76.476633 | 1.918960 | Field Work |
| Colombia | Huila, San Agustin | -76.609966 | 1.902293 | Field Work |
| Colombia | Huila, San Agustin | -76.484966 | 1.893960 | Field Work |
| Colombia | Huila, San Agustin | -76.418299 | 1.893960 | Field Work |
| Colombia | Huila, San Agustin | -76.476633 | 1.877293 | Field Work |
| Colombia | Huila, San Agustin | -76.501633 | 1.868960 | Field Work |
| Colombia | Huila, San Agustin | -76.451633 | 1.868960 | Field Work |
| Colombia | Huila, San Agustin | -76.509966 | 1.860627 | Field Work |
| Colombia | Huila, San Agustin | -76.493299 | 1.860627 | Field Work |
| Colombia | Huila, San Agustin | -76.451633 | 1.860627 | Field Work |
| Colombia | Huila, San Agustin | -76.426633 | 1.852293 | Field Work |
| Colombia | Huila, San Agustin | -76.343299 | 1.827293 | Field Work |
| Colombia | Huila, Santa Maria | -75.734966 | 2.868960 | Field Work |
| Colombia | Huila, Teruel | -75.784966 | 2.868960 | Field Work |
| Colombia | Huila, Teruel | -75.684966 | 2.852293 | Field Work |
| Colombia | Huila, Teruel | -75.693299 | 2.810627 | Field Work |
| Colombia | Nariño, Buesaco | -77.118299 | 1.202293 | Field Work |
| Colombia | Nariño, Funes | -77.334966 | 0.943960 | Field Work |
| Colombia | Nariño, Pasto | -77.126633 | 1.027293 | Field Work |
| Colombia | Nariño, Pasto | -77.209966 | 1.018960 | Field Work |
| Colombia | Nariño, Pasto | -77.301633 | 0.977293 | Field Work |
| Colombia | Nariño, Pasto | -77.226633 | 0.968960 | Field Work |
| Colombia | Nariño, Pasto | -77.118299 | 0.935627 | Field Work |
| Colombia | Nariño, Puerres | -77.368299 | 0.885627 | Field Work |
| Colombia | Putumayo, Orito | -77.043299 | 0.943960 | Field Work |
| Colombia | Putumayo, Orito | -76.993299 | 0.902293 | Field Work |
| Colombia | Putumayo, San Francisco | -76.826633 | 1.285627 | Field Work |
| Colombia | Putumayo, San Francisco | -76.826633 | 1.152293 | Field Work |
| Colombia | Quindío, Salento | -75.468299 | 4.635627 | Field Work |
| Colombia | Risaralda, Pereira | -75.484966 | 4.718960 | Field Work |
| Colombia | Risaralda, Pereira | -75.551632 | 4.702294 | Field Work |
| Colombia | Risaralda, Pereira | -75.526632 | 4.702294 | Field Work |
| Colombia | Risaralda, Pereira | -75.576632 | 4.693960 | Field Work |
| Colombia | Risaralda, Pereira | -75.493299 | 4.693960 | Field Work |
| Colombia | Risaralda, Pereira | -75.501632 | 4.677294 | Field Work |
| Colombia | Risaralda, Pereira | -75.493299 | 4.677294 | Field Work |
| Colombia | Risaralda, Santa Rosa de Cabal | -75.543299 | 4.885627 | Field Work |
| Colombia | Risaralda, Santa Rosa de Cabal | -75.509966 | 4.818960 | Field Work |
| Colombia | Risaralda, Santa Rosa de Cabal | -75.484966 | 4.818960 | Field Work |
| Colombia | Risaralda, Santa Rosa de Cabal | -75.501632 | 4.802294 | Field Work |
| Colombia | Risaralda, Santa Rosa de Cabal | -75.493299 | 4.802294 | Field Work |
| Colombia | Risaralda, Santa Rosa de Cabal | -75.526632 | 4.785627 | Field Work |
| Colombia | Risaralda, Santa Rosa de Cabal | -75.509966 | 4.743960 | Field Work |
| Colombia | Risaralda, Santa Rosa de Cabal | -75.576632 | 4.727294 | Field Work |
| Colombia | Tolima, Anzoátegui | -75.326632 | 4.627294 | Field Work |
| Colombia | Tolima, Cajamarca | -75.468299 | 4.502294 | Field Work |
| Colombia | Tolima, Cajamarca | -75.451632 | 4.468960 | Field Work |
| Colombia | Tolima, Chaparral | -75.701632 | 3.943960 | Field Work |
| Colombia | Tolima, Chaparral | -75.793299 | 3.918960 | Field Work |
| Colombia | Tolima, Chaparral | -75.668299 | 3.918960 | Field Work |
| Colombia | Tolima, Chaparral | -75.818299 | 3.852294 | Field Work |
| Colombia | Tolima, Chaparral | -75.676632 | 3.802294 | Field Work |
| Colombia | Tolima, Ibague | -75.351632 | 4.635627 | Field Work |
| Colombia | Tolima, Ibague | -75.376632 | 4.543960 | Field Work |
| Colombia | Tolima, Ibague | -75.459966 | 4.327294 | Field Work |
| Colombia | Tolima, Planadas | -75.884966 | 3.318960 | Field Work |
| Colombia | Tolima, Planadas | -75.926632 | 3.185627 | Field Work |
| Colombia | Tolima, Planadas | -75.776632 | 3.185627 | Field Work |
| Colombia | Tolima, Planadas | -75.768299 | 3.102293 | Field Work |
| Colombia | Tolima, Rioblanco | -75.809966 | 3.652294 | Field Work |
| Colombia | Tolima, Rioblanco | -75.901632 | 3.493960 | Field Work |
| Colombia | Tolima, Rioblanco | -75.868299 | 3.418960 | Field Work |
| Colombia | Tolima, Roncesvalles | -75.601632 | 4.127294 | Field Work |
| Colombia | Tolima, Roncesvalles | -75.676632 | 4.093960 | Field Work |
| Colombia | Tolima, Roncesvalles | -75.593299 | 3.960627 | Field Work |
| Colombia | Tolima, Rovira | -75.376632 | 4.302294 | Field Work |
| Ecuador | Carchi, Montufar | -77.693299 | 0.568960 | IUCN/SSC TSG-Ecuador |
| Ecuador | Carchi, Montufar | -77.776633 | 0.493960 | IUCN/SSC TSG-Ecuador |
| Ecuador | Chimborazo, Penipe | -78.476633 | -1.456040 | IUCN/SSC TSG-Ecuador |
| Ecuador | Chimborazo, Penipe | -78.401633 | -1.497707 | IUCN/SSC TSG-Ecuador |
| Ecuador | Chimborazo, Penipe | -78.476633 | -1.514373 | IUCN/SSC TSG-Ecuador |
| Ecuador | Chimborazo, Penipe | -78.451633 | -1.522707 | IUCN/SSC TSG-Ecuador |
| Ecuador | Chimborazo, Penipe | -78.426633 | -1.522707 | IUCN/SSC TSG-Ecuador |
| Ecuador | Chimborazo, Penipe | -78.401633 | -1.522707 | IUCN/SSC TSG-Ecuador |
| Ecuador | Loja, Loja | -79.143299 | -4.031040 | IUCN/SSC TSG-Ecuador |
| Ecuador | Morona Santiago, Huamboya | -78.368299 | -1.789373 | IUCN/SSC TSG-Ecuador |
| Ecuador | Morona Santiago, Huamboya | -78.376633 | -1.806040 | IUCN/SSC TSG-Ecuador |
| Ecuador | Morona Santiago, Huamboya | -78.434966 | -1.972707 | IUCN/SSC TSG-Ecuador |
| Ecuador | Morona Santiago, Huamboya | -78.409966 | -1.972707 | IUCN/SSC TSG-Ecuador |
| Ecuador | Morona Santiago, Huamboya | -78.384966 | -1.981040 | IUCN/SSC TSG-Ecuador |
| Ecuador | Morona Santiago, Huamboya | -78.368299 | -1.981040 | IUCN/SSC TSG-Ecuador |
| Ecuador | Morona Santiago, Huamboya | -78.359966 | -1.989373 | IUCN/SSC TSG-Ecuador |
| Ecuador | Napo, El Chaco | -77.851633 | -0.364373 | IUCN/SSC TSG-Ecuador |
| Ecuador | Napo, El Chaco | -77.801633 | -0.289373 | IUCN/SSC TSG-Ecuador |
| Ecuador | Napo, Quijos | -78.218299 | -0.439373 | IUCN/SSC TSG-Ecuador |
| Ecuador | Napo, Quijos | -78.218299 | -0.364373 | IUCN/SSC TSG-Ecuador |
| Ecuador | Napo, Quijos | -78.209966 | -0.406040 | IUCN/SSC TSG-Ecuador |
| Ecuador | Napo, Quijos | -78.201633 | -0.422707 | IUCN/SSC TSG-Ecuador |
| Ecuador | Napo, Quijos | -78.143299 | -0.331040 | Field Work |
| Ecuador | Napo, Quijos | -78.143299 | -0.314373 | Field Work |
| Ecuador | Napo, Quijos | -78.118299 | -0.364373 | Field Work |
| Ecuador | Napo, Quijos | -78.118299 | -0.297707 | Field Work |
| Ecuador | Napo, Quijos | -78.118299 | -0.272707 | Field Work |
| Ecuador | Napo, Quijos | -78.101633 | -0.356040 | Field Work |
| Ecuador | Napo, Quijos | -78.084966 | -0.389373 | IUCN/SSC TSG-Ecuador |
| Ecuador | Napo, Quijos | -78.009966 | -0.447707 | IUCN/SSC TSG-Ecuador |
| Ecuador | Pastaza, Pastaza | -78.043299 | -1.406040 | GBIF-Manis |
| Ecuador | Pichincha, Quito | -78.251633 | -0.214373 | IUCN/SSC TSG-Ecuador |
| Ecuador | Tungurahua, Baños | -78.251633 | -1.214373 | IUCN/SSC TSG-Ecuador |
| Ecuador | Tungurahua, Baños | -78.309966 | -1.222707 | IUCN/SSC TSG-Ecuador |
| Ecuador | Tungurahua, Baños | -78.284966 | -1.222707 | IUCN/SSC TSG-Ecuador |
| Ecuador | Tungurahua, Baños | -78.293299 | -1.239373 | IUCN/SSC TSG-Ecuador |
| Ecuador | Tungurahua, Baños | -78.401633 | -1.356040 | IUCN/SSC TSG-Ecuador |
| Ecuador | Tungurahua, Baños | -78.143299 | -1.364373 | IUCN/SSC TSG-Ecuador |
| Ecuador | Tungurahua, Baños | -78.159966 | -1.381040 | IUCN/SSC TSG-Ecuador |
| Ecuador | Tungurahua, Baños | -78.418299 | -1.397707 | IUCN/SSC TSG-Ecuador |
| Ecuador | Tungurahua, Baños | -78.168299 | -1.397707 | IUCN/SSC TSG-Ecuador |
| Ecuador | Tungurahua, Baños | -78.309966 | -1.422707 | IUCN/SSC TSG-Ecuador |
| Ecuador | Tungurahua, Baños | -78.401633 | -1.447707 | IUCN/SSC TSG-Ecuador |
| Ecuador | Tungurahua, Baños | -78.384966 | -1.447707 | IUCN/SSC TSG-Ecuador |
| Ecuador | Tungurahua, Baños | -78.309966 | -1.447707 | IUCN/SSC TSG-Ecuador |
| Ecuador | Tungurahua, Baños | -78.384966 | -1.472707 | IUCN/SSC TSG-Ecuador |
| Ecuador | Tungurahua, Baños | -78.384966 | -1.489373 | IUCN/SSC TSG-Ecuador |
| Ecuador | Tungurahua, Patate | -78.326633 | -1.231040 | IUCN/SSC TSG-Ecuador |
| Ecuador | Tungurahua, Patate | -78.359966 | -1.247707 | IUCN/SSC TSG-Ecuador |
| Ecuador | Tungurahua, Patate | -78.334966 | -1.247707 | IUCN/SSC TSG-Ecuador |
| Ecuador | Tungurahua, Patate | -78.343299 | -1.256040 | IUCN/SSC TSG-Ecuador |
| Ecuador | Tungurahua, Patate | -78.368299 | -1.264373 | IUCN/SSC TSG-Ecuador |
| Ecuador | Tungurahua, Patate | -78.384966 | -1.272707 | IUCN/SSC TSG-Ecuador |
| Ecuador | Tungurahua, Patate | -78.401633 | -1.306040 | IUCN/SSC TSG-Ecuador |
| Ecuador | Tungurahua, Santiago Pillaro | -78.426633 | -1.197707 | IUCN/SSC TSG-Ecuador |
| Ecuador | Tungurahua, Sn Pedro Pelileo | -78.468299 | -1.456040 | IUCN/SSC TSG-Ecuador |
| Ecuador | Zamora Chinchipe, Yacuambi | -78.984966 | -3.606040 | UTPL |
| Peru | Cajamarca, Cutervo | -79.276633 | -6.147707 | Field Work |
| Peru | Cajamarca, Cutervo | -79.268299 | -6.122707 | Field Work |
| Peru | Cajamarca, Cutervo | -79.243299 | -6.139374 | Field Work |
| Peru | Cajamarca, San Ignacio | -79.326633 | -5.189374 | Field Work |
| Peru | Cajamarca, San Ignacio | -79.284966 | -5.231040 | Field Work |
| Peru | Cajamarca, San Ignacio | -79.276633 | -5.264374 | Field Work |
| Peru | Cajamarca, San Ignacio | -79.276633 | -5.239374 | Field Work |
| Peru | Cajamarca, San Ignacio | -79.243299 | -5.247707 | Field Work |
| Peru | Cajamarca, San Ignacio | -79.034966 | -5.297707 | Field Work |
| Peru | Lambayeque, Ferrenate | -79.301633 | -6.139374 | Field Work |
| Peru | Lambayeque, Ferrenate | -79.301633 | -6.106040 | Field Work |
| Peru | Lambayeque, Ferrenate | -79.293299 | -6.122707 | Field Work |
| Peru | Lambayeque, Ferrenate | -79.284966 | -6.147707 | Field Work |
| Peru | Piura, Ayabaca | -79.484966 | -4.956040 | Field Work |
| Peru | Piura, Ayabaca | -79.451633 | -4.922707 | Field Work |
| Peru | Piura, Huancabamba | -79.459966 | -4.956040 | Field Work |
| Peru | Piura, Huancabamba | -79.443299 | -4.956040 | Field Work |
